# Molecular harvesting with electroporation for tissue profiling

**DOI:** 10.1038/s41598-019-51634-7

**Published:** 2019-10-31

**Authors:** Alexander Golberg, Julia Sheviryov, Oz Solomon, Leon Anavy, Zohar Yakhini

**Affiliations:** 1 Porter School of Environment and Earth Sciences, Tel Aviv University, Tel Aviv, Israel; 2 School of Computer Science, Herzliya Interdisciplinary Center, Herzliya, Israel; 3 Computer Science Department, Technion, Haifa, Israel

**Keywords:** Oncology, Biomedical engineering

## Abstract

Recent developments in personalized medicine are based on molecular measurement steps that guide personally adjusted medical decisions. A central approach to molecular profiling consists of measuring DNA, RNA, and/or proteins in tissue samples, most notably in and around tumors. This measurement yields molecular biomarkers that are potentially predictive of response and of tumor type. Current methods in cancer therapy mostly use tissue biopsy as the starting point of molecular profiling. Tissue biopsies involve a physical resection of a small tissue sample, leading to localized tissue injury, bleeding, inflammation and stress, as well as to an increased risk of metastasis. Here we developed a technology for harvesting biomolecules from tissues using electroporation. We show that tissue electroporation, achieved using a combination of high-voltage short pulses, 50 pulses 500 V cm^−1^, 30 µs, 1 Hz, with low-voltage long pulses 50 pulses 50 V cm^−1^, 10 ms, delivered at 1 Hz, allows for tissue-specific extraction of RNA and proteins. We specifically tested RNA and protein extraction from excised kidney and liver samples and from excised HepG2 tumors in mice. Further *in vivo* development of extraction methods based on electroporation can drive novel approaches to the molecular profiling of tumors and of tumor environment and to related diagnosis practices.

## Introduction

Personalized medicine is the optimization of care on an individual basis. Personalized medicine, based on molecular profiles of tumors and other tissues, has greatly developed over recent decades. In cancer therapy and care, a clear potential in several cases was demonstrated for the personalized approach as compared to traditional therapies^1–3^. Accurate diagnosis is a critical component of this approach. An important component of molecular diagnostics in diseased tissues, including tumors, is the profiling of DNA, RNA, proteins or metabolites, to identify molecular biomarkers that are predictive of tumor type^4–7^ and of patient response^8,9^. To enable tumor profiling, current methods use liquid^10^ or tissue biopsy. Tissue biopsy involves resection of a small tissue sample, a procedure which leads to localized tissue injury, bleeding inflammation, neural damage, fracture, and stress^11,12^, all increase the potential for tumor growth and metastasis^13–15^. The impact of this stress on tumor development is not well understood^15^. In addition, only a few biopsies can be performed at a time, limiting the scope of the spatial mapping of the sampled site, and leading to misdiagnosis if the tumor is missed or if a less relevant clonal sub-population is probed. Furthermore, some studies even concluded that due to tumor heterogeneity, information from a single biopsy is not sufficient for guiding treatment decisions in prostate cancer^16,17^.

Indeed, recent literature identified the limited support, by current technology, for characterizing tumor molecular heterogeneity^18^, as a major limitation of the personalized medicine approach in cancer^19^. Significant genomic evolution often occurs during cancer progression, creating variability within primary tumors as well as between the primary tumors and metastases^17,20,21^. Indeed, recent studies show that a positive result (both successful biopsy and a decisive detection of markers) appear to reliably indicate the presence of the high-risk disease^16^. However, a negative result does not reliably rule out the presence of high-risk clones. This is partly because a harvested tissue sample may not capture the most aggressive clone of a given tumor or tumor site^16^. Despite the significant improvement of molecular characterization technologies, during recent decades, thanks to the introduction of new high-resolution sequencing and bioinformatics methods, these technologies remain limited by tissue sampling methods^17,22^. Thus, tissue sampling remains a critical limitation to personalized medicine^16,17,23^. Therefore, new approaches to molecularly probing and characterizing several regions in the tumor, termed molecular cartography, are called for^24^.

To extend the state of the art of technologies that enable precision therapy, we developed a novel approach for tissue sampling with molecular biopsy using electroporation^25^. Electroporation-based technologies have been successfully used to non-thermally irreversibly and reversibly change permeabilization of the cell membrane *in vivo*, enabling a wide set of applications ranging from tumor ablation to targeted delivery of molecules to tissues^25^. The important aspect of electroporation-based technologies, which distinguishes it from other methods is that the change inflicted on tissue permeabilization is chemical-free and non-thermal^26,27^, features that can help preserve the properties of the delivered or extracted molecules^28,29^. We and others have previously developed protocols for targeted delivery of electric fields to tissues to induce focused electroporation at the predetermined region in organs^30–35^. More recently, we have shown that electroporation technologies selectively extract proteins and ash from biomass^36–38^. Although electroporation has been used to deliver molecules to tissues and to ablate multiple tumors and metastatic sites, it has not so far, to the best of our knowledge, been proposed for extracting molecules for tissue molecular profiling, including tumors.

The goal of this work is to test electroporation based harvesting (e-harvesting) protocols and assess the molecular profiles of RNA and proteins obtained through this procedure. In particular, we show that transcriptomic and proteomic profiles obtained by e-harvesting from excised HepG2 liver tumor samples in mice, normal murine liver and normal murine kidney, are tissue-specific and that they align with literature molecular information related to these samples. Literature measurement of molecular differences between these types of samples was obtained using standard extraction methods. This new approach to tissue characterization is substantially different from needle or other excision biopsies (with the associated risks as described above), as well as from liquid biopsy (which only sees an average profile and can not provide sub-clonal information). Our approach, if and when used in combination with *in-situ* electrodes^39–41^, can potentially provide access to tumor molecular markers from organs harboring tumors even when the exact location of the tumors is not known. Furthermore, in the future, it could potentially lead to enabling multiple sampling and thereby to spatial molecular cartography of tissues.

## Results

### Transcriptomics and proteomics differences detected with e-harvesting in mouse liver and kidney

**Figure 1:**
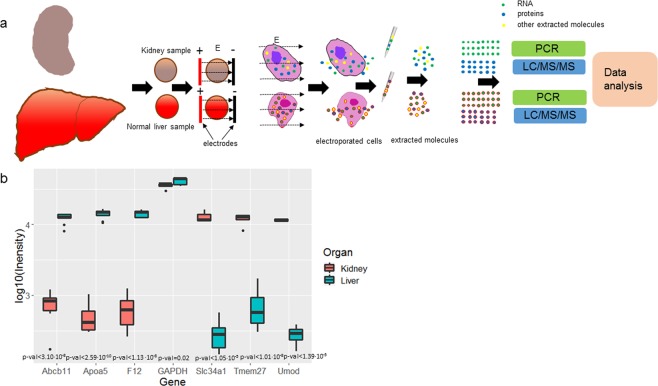
(**a**) Protocol for molecular harvesting by electroporation (e-harvesting) from mouse normal liver and kidney samples. (**b**) Differential expression of genes detected in e-harvested samples of mouse liver and kidney; N = 6.

We tested the protocol for e-harvesting from the normal liver and normal kidney as shown in Fig. 1a. Our sample set consisted of 27 normal liver and 18 normal kidney samples from 3 mice.

Using qPCR on RNA extracted from kidney samples by e-harvesting (see Methods) we found that RNA encoding for Tmem27, Umod, and Slc34a1 were significantly overexpressed (p-val < 10^−4^) compared to RNA for these genes extracted from the liver (Figs 1b and S1). Furthermore, Apoa5, F12, and Abcb11 genes were significantly (p-val < 10^−4^) higher in the e-extracts from the liver than in the e-extracts from the kidney (Figs 1b and S1). These results are consistent with the literature used to select these genes for testing (see Methods).

Proteins were also extracted from the samples, following e-harvesting, and then profiled using unlabeled proteomics by LC/MS-MS (see Methods). Using proteomics, we identified 2078 proteins in both kidney and liver (Tables S1 and S2). Using GOrilla^42^ we performed gene ontology analysis for the associated genes, working with the ranked list of differently expressed proteins, annotated to the mouse genome (Table S3). We also analyze the molecular weight (MW) of the extracted proteins and observe a lognormal distribution (Table 1) as further described in Supplementary Information 1.

**Table 1:** Descriptive statistics of molecular weights of proteins extracted from tissues with electroporation.

Tissue	Min	1^st^ Qu.	Median	Mean	3^rd^ Qu.	Max
Kidney	2.79	11.99	18.01	25.21	31.53	106.25
Liver	3.81	18.38	30.53	36.38	48.35	272.43
HepG2	3.82	18.92	32.19	40.26	50.88	394.46

iBAQ > 10^7^.

**Figure 2:**
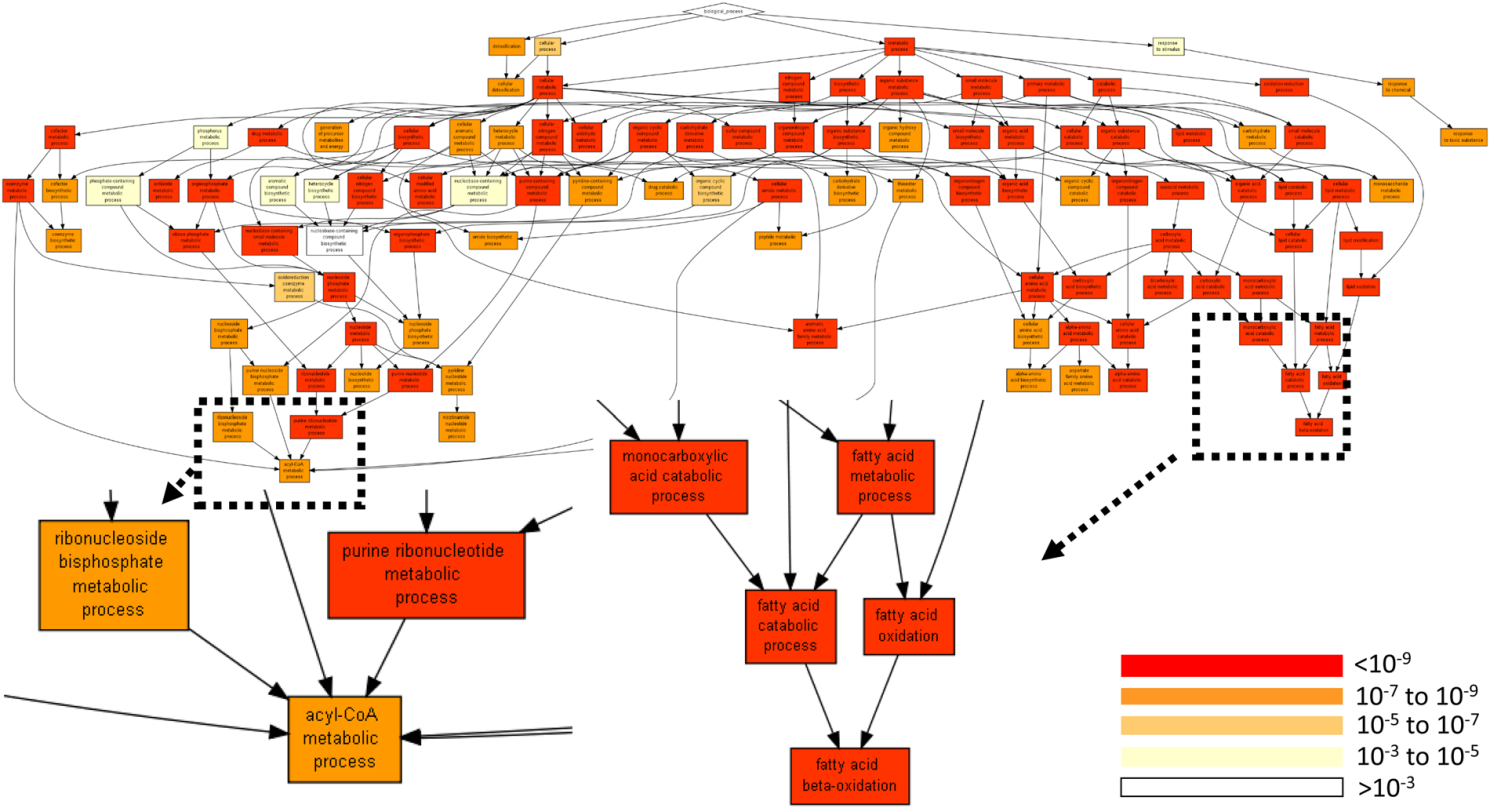
Annotation of the identified proteins to processes. The annotation applies to all identified proteins (Tables S1 and S2) and is performed by GOrilla^42^ using a list ranked by (LFQ_liver-LFQ_Kidney) values.

Analysis of gene ontology terms by processes showed that the processes of small molecule metabolism, organic acid metabolism, drug metabolism, and fatty acid metabolism were more active in the liver than in the kidney (Fig. 2, Tables 2 and S4).

**Table 2:** Gene ontology by a process of the differently expressed proteins in the liver and the kidney extracted with electroporation mapped with GOrilla^42^.

GO term	Description	P-value*	FDR q-value**	Enrichment (N, B, n, b)***
GO:0044281	small molecule metabolic process	2.34E-50	1.90E-46	2.72 (1731, 323, 333, 169)
GO:0006082	organic acid metabolic process	6.13E-44	2.48E-40	3.40 (1731, 209, 285, 117)
GO:0043436	oxoacid metabolic process	7.64E-43	2.06E-39	3.39 (1731, 206, 285, 115)
GO:0019752	carboxylic acid metabolic process	1.87E-41	3.78E-38	3.36 (1731, 204, 285, 113)
GO:0055114	oxidation-reduction process	1.13E-37	1.84E-34	2.37 (1731, 218, 510, 152
GO:0044282	small molecule catabolic process	8.09E-36	1.09E-32	4.49 (1731, 91, 292, 69)
GO:0008152	metabolic process	4.76E-35	5.51E-32	1.42 (1731, 914, 547, 411)
GO:0016054	organic acid catabolic process	2.93E-30	2.97E-27	4.72 (1731, 72, 285, 56
GO:0046395	carboxylic acid catabolic process	2.93E-30	2.64E-27	4.72 (1731, 72, 285, 56)
GO:0071704	organic substance metabolic process	6.56E-30	5.32E-27	1.46 (1731, 816, 524, 360)
GO:0051186	cofactor metabolic process	6.16E-27	4.54E-24	2.60 (1731, 145, 460, 100)
GO:0032787	monocarboxylic acid metabolic process	6.35E-27	4.29E-24	2.74 (1731, 116, 480, 8)
GO:0017144	drug metabolic process	3.02E-26	1.88E-23	2.60 (1731, 143, 457, 98)
GO:0044237	cellular metabolic process	5.98E-26	3.46E-23	1.44 (1731, 794, 524, 346)
GO:0009056	catabolic process	2.33E-24	1.26E-21	2.43 (1731, 279, 294, 115)

*‘P-value’ is the enrichment p-value computed according to the mHG or HG model. This p-value is not corrected for multiple testing of 731 GO terms. **‘FDR q-value’ is the correction of the above p-value for multiple testing using the Benjamini and Hochberg (1995) method. Namely, for the i^th^ term (ranked according to p-value) the FDR q-value is (p-value * a number of GO terms)/i. ***Enrichment (N, B, n, b) is defined as follows: N - is the total number of genes. B - is the total number of genes associated with a specific GO term. n - is the number of genes in the top of the user’s input list or in the target set when appropriate. b - is the number of genes in the intersection. Enrichment = (b/n)/(B/N).

Analysis of function categories shows multiple significant functional differences between the liver and the kidney, including catalytic activity, drug binding, and fatty-acyl-CoA binding, lyase activity^43^, oxidoreductase^44^ activities, which are all expressed higher in the liver (Tables 3 and S5).

**Table 3:** Gene ontology by a function of the differently expressed proteins in the liver and the kidney extracted with electroporation mapped with GOrilla^42^.

GO term	Description	P-value*	FDR q-value**	Enrichment (N, B, n, b)***
GO:0003824	catalytic activity	4.64E-50	9.28E-47	1.75 (1731, 672, 480, 326)
GO:0016491	oxidoreductase activity	1.23E-37	1.23E-34	2.78 (1731, 197, 398, 126)
GO:0048037	cofactor binding	2.47E-27	1.64E-24	2.56 (1731, 141, 484, 101)
GO:0050662	coenzyme binding	9.02E-21	4.5E-18	3.12 (1731, 93, 376, 63)
GO:0036094	small molecule binding	2.65E-14	1.06E-11	2.13 (1731, 336, 230, 95)
GO:0031406	carboxylic acid binding	5.16E-12	1.72E-9	3.58 (1731, 49, 316, 32)
GO:0016829	lyase activity	1.47E-11	4.21E-9	3.50 (1731, 46, 333, 31)
GO:0043177	organic acid binding	2.7E-11	6.74E-9	3.44 (1731, 51, 316, 32)
GO:0042802	identical protein binding	6.05E-11	1.34E-8	2.46 (1731, 299, 127, 54)
GO:1901265	nucleoside phosphate binding	2.98E-9	5.95E-7	1.43 (1731, 257, 743, 158)
GO:0000166	nucleotide binding	2.98E-9	5.41E-7	1.43 (1731, 257, 743, 158)
GO:0016616	oxidoreductase activity, acting on the CH-OH group of donors, NAD or NADP as acceptor	4.38E-9	7.3E-7	4.04 (1731, 43, 229, 23)
GO:0016836	hydro-lyase activity	5.24E-9	8.05E-7	6.79 (1731, 17, 195, 13)
GO:0051287	NAD binding	6.27E-9	8.94E-7	3.31 (1731, 29, 415, 23)
GO:0003735	structural constituent of ribosome	1.69E-8	2.25E-6	2.67 (1731, 35, 518, 28)

*‘P-value’ is the enrichment p-value computed according to the mHG or HG model. This p-value is not corrected for multiple testing of 731 GO terms. **‘FDR q-value’ is the correction of the above p-value for multiple testing using the Benjamini and Hochberg (1995) method. Namely, for the i^th^ term (ranked according to p-value) the FDR q-value is (p-value * a number of GO terms)/i. ***Enrichment (N, B, n, b) is defined as follows: N - is the total number of genes. B - is the total number of genes associated with a specific GO term. n - is the number of genes in the top of the user’s input list or in the target set when appropriate. b - is the number of genes in the intersection. Enrichment = (b/n)/(B/N).

Analysis by component showed large differences in mitochondrion related proteins extracted from the liver vs kidney (Tables 4 and S6).

**Table 4:** Gene ontology by a component of the differently expressed proteins in the liver and the kidney extracted with electroporation mapped with GOrilla^42^.

GO term	Description	P-value*	FDR q-value**	Enrichment (N, B, n,b)***
GO:0005739	mitochondrion	4.63E-37	5.23E-34	2.00 (1731, 429, 432, 214
GO:0044429	mitochondrial part	9.3E-20	5.26E-17	1.90 (1731, 235, 542, 140)
GO:0044444	cytoplasmic part	4.7E-16	1.77E-13	1.24 (1731, 1195, 428, 366)
GO:0005743	mitochondrial inner membrane	4.81E-16	1.36E-13	1.96 (1731, 144, 612, 100)
GO:0031966	mitochondrial membrane	9.05E-16	2.05E-13	2.22 (1731, 175, 401, 90)
GO:0043209	myelin sheath	7.47E-15	1.41E-12	2.30 (1731, 78, 597, 62)
GO:0019866	organelle inner membrane	2.18E-14	3.52E-12	1.97 (1731, 149, 559, 95)
GO:0043233	organelle lumen	1.2E-9	1.69E-7	2.30 (1731, 94, 408, 51)
GO:0070013	intracellular organelle lumen	1.2E-9	1.5E-7	2.30 (1731, 94, 408, 51)
GO:0031974	membrane-enclosed lumen	1.2E-9	1.35E-7	2.30 (1731, 94, 408, 51)
GO:0022627	cytosolic small ribosomal subunit	1.84E-9	1.89E-7	4.06 (1731, 20, 384, 18)
GO:0042579	microbody	3.44E-9	3.24E-7	2.61 (1731, 47, 480, 34)
GO:0005777	peroxisome	9.61E-9	8.36E-7	2.59 (1731, 46, 480, 33)
GO:0005759	mitochondrial matrix	1.65E-8	1.33E-6	2.20 (1731, 48, 623, 38)
GO:0031090	organelle membrane	3.01E-7	2.27E-5	1.56 (1731, 296, 401, 107)

*‘P-value’ is the enrichment p-value computed according to the mHG or HG model. This p-value is not corrected for multiple testing of 731 GO terms. **‘FDR q-value’ is the correction of the above p-value for multiple testing using the Benjamini and Hochberg (1995) method. Namely, for the i^th^ term (ranked according to p-value) the FDR q-value is (p-value * a number of GO terms)/i. ***Enrichment (N, B, n, b) is defined as follows: N - is the total number of genes. B - is the total number of genes associated with a specific GO term. n - is the number of genes in the top of the user’s input list or in the target set when appropriate. b - is the number of genes in the intersection. Enrichment = (b/n)/(B/N).

We further compared the proteomic profiles and differences we obtained using the e-harvesting protocol to the literature^45^. Literature differential proteomics results were obtained using standard extraction protocols. We generated a list of differently expressed proteins from our proteomics measurements as well as one from the mouse protein atlas and compared the ranking of 1496 proteins common to both lists using the min-min Hypergeometric statistics, m^2^HG^46–48^ (Table S7). We observed that the two ranked lists are mutually enriched, with an m^2^HG p-value < 1E-40. In the top 428 proteins in the ranking obtained using e-harvesting we observe 150 of the top 200 proteins in the ranking obtained from the literature.

### Transcriptomics and proteomics differences detected with e-harvesting in HepG2 human tumor model compared to the normal liver in the mouse

**Figure 3:**
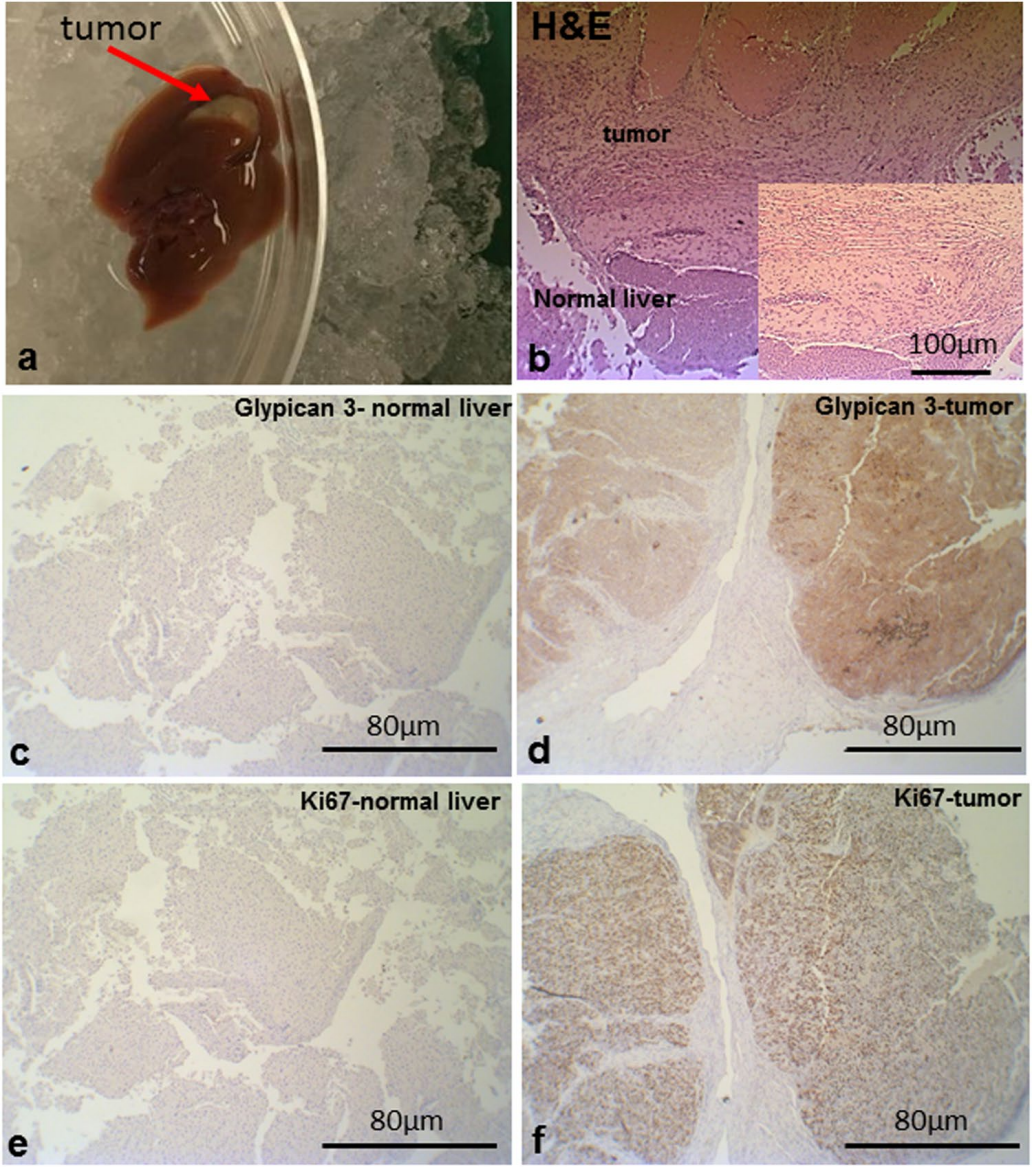
(**a**) Excised liver with the HepG2 tumor. (**b**) Hematoxylin and eosin (H&E) staining of the mouse liver samples with HepG2 tumor. Glypican 3 expression in the normal liver section (**c**) and in the section with GepG2 tumor (**d**) Ki67 expression in the normal liver section (**e**) and in the section with GepG2 tumor (**f**).

We next tested our e-harvesting protocol by profiling HepG2 tumors grafted into murine livers (Fig. 3a). Histological examination clearly shows abnormal cells and tissue structures at the tumor area vs a normal liver structure (Fig. 3b). Tumors area showed an expected higher expression of Glypican 3^49^ and Ki67^50^. For the comparison to normal mouse liver, we profiled 7 tumor samples and 14 normal mouse liver samples from 5 mice.

**Figure 4:**
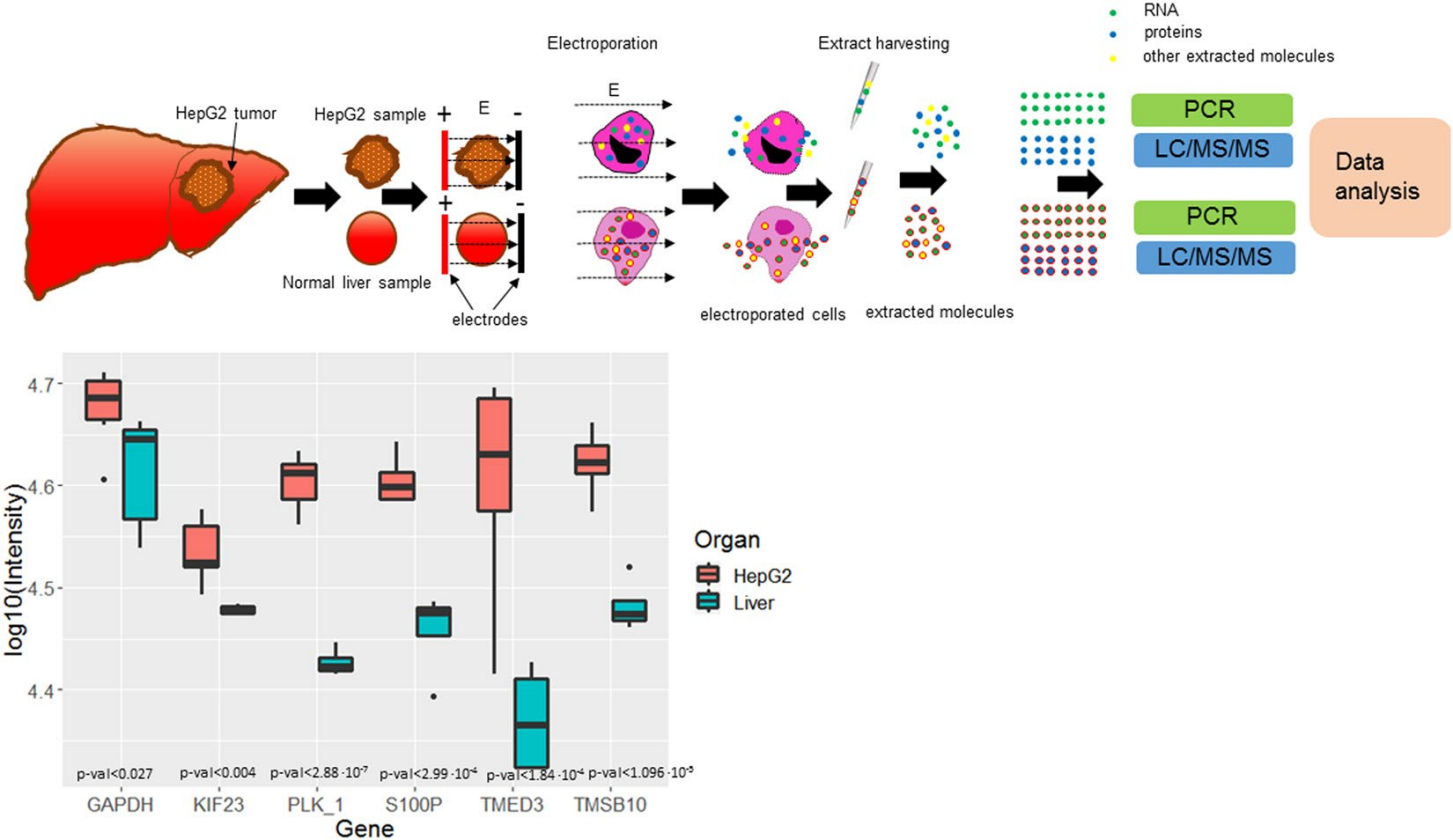
(**a**) Protocol for electroporation based molecular harvesting from normal livers and from HepG2 tumor models in mouse. (**b**) Differential expression of genes detected with RNA extracted using the proposed e-harvesting protocol from mouse liver and from HepG2; N = 6.

We found that in the e-extracts from the HepG2 liver model in mice, RNA encoding for PLK_1, S100P, TMED3, TMSB10, and KIF23 were expressed at significantly higher levels than RNA for these genes e-extracted from the normal liver (Figs 4 and S2).

Proteins were also extracted from the samples, following e-harvesting, and then profiled using unlabeled proteomics by LC/MS-MS (see Methods) (Table S8). For 2782 proteins from the HepG2 and the normal liver, identified using this approach, we performed gene ontology analysis with the associated genes (on the ranked list of differently expressed proteins, Table S9) using GOrilla^42^, annotating the ranked gene list to the mouse genome. We also analyze the molecular weight (MW) of the extracted proteins and observe a lognormal distribution (Table 1) as further described in Supplementary Information 1.

**Figure 5:**
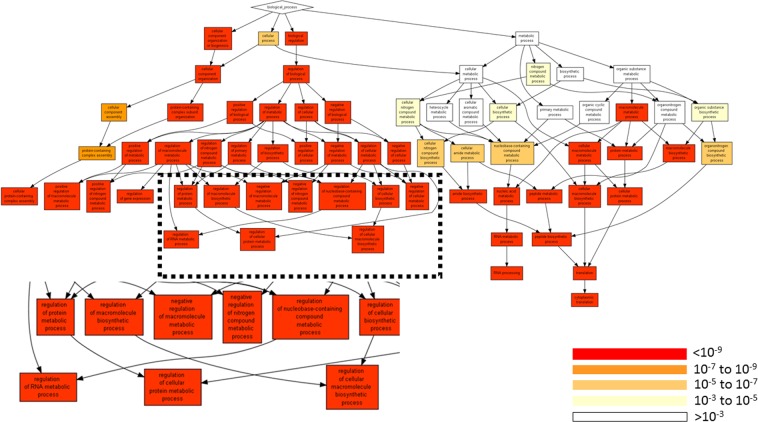
Annotation of the identified proteins to process GO terms. The annotation was done on all identified proteins and is performed by GOrilla^42^ using a list ranked by (LFQ_HepG2-LFQ_Liver) values.

Analysis of gene ontology terms by processes showed that the processes of macromolecules metabolism, nucleic acid metabolism, regulation of cellular processes and macromolecule biosynthesis were higher in the HepG2 than in the normal liver (Fig. 5, Tables 5 and S10).

**Table 5:** Gene ontology by a process of the differently expressed proteins in the HepG2 the normal liver extracted with electroporation mapped with GOrilla^42^.

GO term	Description	P-value*	FDR q-value**	Enrichment (N, B, n, b)***
GO:0043170	macromolecule metabolic process	2.47E-23	2.22E-19	1.36 (2589, 919, 992, 478)
GO:0044260	cellular macromolecule metabolic process	1.98E-21	8.93E-18	1.44 (2589, 648, 992, 358)
GO:0090304	nucleic acid metabolic process	5.93E-20	1.78E-16	1.64 (2589, 334, 992, 210)
GO:0050789	regulation of biological process	7.73E-20	1.74E-16	1.25 (2589, 1301, 968, 607)
GO:0051171	regulation of nitrogen compound metabolic process	1.08E-19	1.94E-16	1.57 (2589, 695, 639, 269)
GO:0050794	regulation of cellular process	1.55E-19	2.33E-16	1.27 (2589, 1202, 970, 570)
GO:0060255	regulation of macromolecule metabolic process	1.86E-19	2.4E-16	1.52 (2589, 718, 689, 291)
GO:0080090	regulation of primary metabolic process	8.83E-19	9.95E-16	1.54 (2589, 731, 639, 277)
GO:0006412	translation	1.07E-16	1.07E-13	2.83 (2589, 161, 398, 70)
GO:0048519	negative regulation of biological process	1.57E-16	1.41E-13	1.45 (2589, 720, 759, 306)
GO:0034645	cellular macromolecule biosynthetic process	2.77E-16	2.27E-13	2.50 (2589, 231, 381, 85)
GO:0009059	macromolecule biosynthetic process	4.38E-16	3.29E-13	2.45 (2589, 241, 381, 87)
GO:0048523	negative regulation of cellular process	4.78E-16	3.31E-13	1.47 (2589, 655, 759, 283)
GO:0043043	peptide biosynthetic process	5.81E-16	3.74E-13	2.76 (2589, 165, 398, 70)
GO:0010468	regulation of gene expression	7.68E-16	4.61E-13	1.53 (2589, 480, 846, 240)

*‘P-value’ is the enrichment p-value computed according to the mHG or HG model. This p-value is not corrected for multiple testing of 731 GO terms. **‘FDR q-value’ is the correction of the above p-value for multiple testing using the Benjamini and Hochberg (1995) method. Namely, for the i^th^ term (ranked according to p-value) the FDR q-value is (p-value * a number of GO terms)/i. ***Enrichment (N, B, n, b) is defined as follows: N - is the total number of genes. B - is the total number of genes associated with a specific GO term. n - is the number of genes in the top of the user’s input list or in the target set when appropriate. b - is the number of genes in the intersection. Enrichment = (b/n)/(B/N).

Analysis of the function terms shows multiple significant functional differences between the HepG2 as compared to the normal murine liver, including nucleic acid binding, protein binding, and oxygen binding, all expressed higher in the tumor (Tables 6 and S11).

**Table 6:** Gene ontology by function of the differently expressed proteins in the HepG2 the normal liver extracted with electroporation mapped with GOrilla^42^.

GO term	Description	P-value*	FDR q-value**	Enrichment (N, B, n, b)***
GO:0003676	nucleic acid binding	5.63E-39	1.46E-35	1.73 (2589, 461, 991, 306)
GO:0003723	RNA binding	5.41E-31	7.02E-28	1.79 (2589, 342, 981, 232)
GO:0005515	protein binding	2.13E-28	1.85E-25	1.26 (2589, 1435, 999, 696)
GO:0003735	structural constituent of ribosome	5.96E-24	3.87E-21	4.18 (2589, 95, 372, 57)
GO:0005198	structural molecule activity	1.29E-21	6.73E-19	2.89 (2589, 191, 398, 85)
GO:0005488	binding	6.16E-19	2.67E-16	1.12 (2589, 2046, 999, 884)
GO:0003729	mRNA binding	2.28E-15	8.47E-13	2.53 (2589, 98, 679, 65)
GO:0003677	DNA binding	1.74E-14	5.66E-12	1.81 (2589, 163, 991, 113)
GO:0044877	protein-containing complex binding	1.19E-13	3.44E-11	1.60 (2589, 324, 890, 178)
GO:0019899	enzyme binding	1.34E-12	3.49E-10	1.82 (2589, 463, 375, 122)
GO:0019843	rRNA binding	3.13E-12	7.39E-10	4.73 (2589, 33, 398, 24)
GO:1901363	heterocyclic compound binding	9.98E-12	2.16E-9	1.23 (2589, 973, 992, 460)
GO:0097159	organic cyclic compound binding	3.02E-11	6.04E-9	1.22 (2589, 995, 992, 467)
GO:0008092	cytoskeletal protein binding	1.98E-10	3.68E-8	1.76 (2589, 198, 756, 102)
GO:0043565	sequence-specific DNA binding	2.18E-10	3.77E-8	2.18 (2589, 73, 845, 52)

*‘P-value’ is the enrichment p-value computed according to the mHG or HG model. This p-value is not corrected for multiple testing of 731 GO terms. **‘FDR q-value’ is the correction of the above p-value for multiple testing using the Benjamini and Hochberg (1995) method. Namely, for the i^th^ term (ranked according to p-value) the FDR q-value is (p-value * a number of GO terms)/i. ***Enrichment (N, B, n, b) is defined as follows: N - is the total number of genes. B - is the total number of genes associated with a specific GO term. n - is the number of genes in the top of the user’s input list or in the target set when appropriate. b - is the number of genes in the intersection. Enrichment = (b/n)/(B/N).

Analysis by component showed large differences in the levels of proteins from cytosolic parts, from the protein-containing complexes and from the ribonucleoprotein complex extracted from HepG2 vs the normal liver (Tables 7 and S12).

**Table 7:** Gene ontology by component of the differently expressed proteins in the HepG2 the normal liver extracted with electroporation mapped with GOrilla^42^.

GO term	Description	P-value*	FDR q-value**	Enrichment (N, B, n, b)***
GO:0044445	cytosolic part	3.36E-41	4.19E-38	4.74 (2589, 119, 372, 81)
GO:0032991	protein-containing complex	5.55E-40	3.46E-37	1.40 (2589, 1107, 994, 593)
GO:1990904	ribonucleoprotein complex	1.08E-31	4.49E-29	2.16 (2589, 323, 662, 178)
GO:0005634	nucleus	2.43E-31	7.59E-29	1.41 (2589, 922, 991, 498)
GO:0043232	intracellular non-membrane-bounded organelle	1.04E-25	2.58E-23	1.51 (2589, 597, 991, 345)
GO:0043228	non-membrane-bounded organelle	1.34E-25	2.79E-23	1.51 (2589, 602, 991, 347)
GO:0044391	ribosomal subunit	4.82E-24	8.59E-22	3.92 (2589, 110, 372, 62)
GO:0044428	nuclear part	2.5E-23	3.9E-21	1.43 (2589, 720, 992, 394)
GO:0005840	ribosome	1.87E-22	2.59E-20	3.83 (2589, 109, 372, 60)
GO:0005737	cytoplasm	9.86E-22	1.23E-19	1.25 (2589, 1339, 973, 629)
GO:0022625	cytosolic large ribosomal subunit	3.54E-21	4.01E-19	4.58 (2589, 43, 500, 38)
GO:0045202	synapse	1E-17	1.04E-15	2.54 (2589, 192, 456, 86)
GO:0022627	cytosolic small ribosomal subunit	4.33E-17	4.16E-15	5.72 (2589, 35, 362, 28)
GO:0005681	spliceosomal complex	1.39E-13	1.24E-11	2.12 (2589, 80, 975, 64)
GO:0097458	neuron part	1.42E-13	1.18E-11	1.60 (2589, 321, 840, 167)

*‘P-value’ is the enrichment p-value computed according to the mHG or HG model. This p-value is not corrected for multiple testing of 731 GO terms. **‘FDR q-value’ is the correction of the above p-value for multiple testing using the Benjamini and Hochberg (1995) method. Namely, for the i^th^ term (ranked according to p-value) the FDR q-value is (p-value * a number of GO terms)/i. ***Enrichment (N, B, n, b) is defined as follows: N - is the total number of genes. B - is the total number of genes associated with a specific GO term. n - is the number of genes in the top of the user’s input list or in the target set when appropriate. b - is the number of genes in the intersection. Enrichment = (b/n)/(B/N).

## Discussion

In this work, we tested molecular harvesting by electroporation from normal tissue samples as well as from tumor samples and assessed the molecular profiles of RNA and proteins obtained by this procedure. We showed that e-harvested RNA and proteins from HepG2 liver tumor in mouse liver, normal murine liver, and normal murine kidney are tissue-specific. These results suggest that e-extraction could be used for sample handling that can lead to informative molecular signatures.

Molecular extraction is a starting point in any molecular diagnostic assay^51^. The procedures include tissue disruption, cell lysis, sample pre-fractionation, and separation^52^. Although chemical, enzymatic and mechanical methods, including grinding, shearing, beating, and shocking to obtain tissue permeabilization for molecular extraction are well developed^53,54^, the extraction of molecules for analysis and for diagnosis at the point of care is still very challenging^55^. In addition, most of the current methods are very low-throughput, require individual sample manipulation and are not suitable for rapid extractions. The later is often required when the sample is sensitive and degrades rapidly^54,56^. To address these challenges, electric fields have been investigated in the recent decade as potentially supporting improved molecular extraction^54^. High-voltage, pulsed electric fields that lead to tissue electroporation is a specific example of these emerging technologies. Previous works demonstrated the extraction of genomic DNA^57^, RNA^58^, and proteins^59^ from cells and tissues by electroporation. However, to the best of our knowledge, no previous work investigated molecular signatures, nor did any previous work perform differentiation expression analysis, as shown in this work.

The e-extract of the kidney contained higher expression Tmem27, Umod and Slc34a1 and the extract from the liver contained higher expression Apoa5, F12, and Abcb11 (Fig. 1), which aligns with literature reports^60^. This demonstration of differential expression measured in e-extracts was also corroborated with the study on the RNA extraction from the HepG2 tumor model in the mice liver. We found that in RNA e-extracted from the HepG2 liver model in mice RNA molecules encoding for PLK_1, S100P, TMED3, TMSB10, and KIF23 were significantly overexpressed as compared to RNA molecules of these genes e-extracted from the normal liver (Fig. 4). The higher expression of these genes in HepG2 was shown previously in the RNA-seq data from these tumors^61,62^. Taken together, our data suggest that electroporation extraction maintains some of the relative expression of RNA and allows for the faithful detection of differential expression between kidney, liver, and tumor in the liver.

However, the impact of the pulsed electric fields on RNA integrity, which is important for the downstream analysis^63^ is still not known and is the subject of current invetigation^64,65^. Importantly, our previous work on the impact of electroporation type pulsed electric fields on the supercoiled DNA showed that electric fields cause the change in the size of the supercoiled DNA molecules, probably by nicking^29^. Future protocols development should focus on the extraction of RNA with high integrity, which could be used for sequencing.

The proteomic analysis of the electroporation extracted samples showed that proteins extracted from tissues are aligned with literature^45^ in terms of relative tissue abundance (Figs 2 and 5). Gene Ontology (GO) analysis performed on the ranked lists of the extracted proteins showed significant differences for process, function, and component terms of proteins extracted from the kidney, liver and HepG2 tumor model in the murine liver. Our previous studies on proteins extraction with electroporation from the chicken breast muscle also showed that using Gene Ontology analysis antioxidant function of the extracted proteins could be predicted^59^. The current work expanded that preliminary observation and shows that the extracted proteins are tissue-specific and potentially could allow differential expression measurements in various tissues including tumors. In the aforementioned previous work, we also showed that electroporation leads to selective extraction of proteins and ash from the seaweed^36,37^. Therefore, future studies of e-harvesting in tissue samples should determine the properties of the extractable proteins^37,38^. These properties depend on the tissue structure, using pulsed electric fields protocols and the extraction solvent^66^. This work does not address the physicochemical properties of the extracted proteins, which predicate their extractability and solubility^67^ and additional studies in this direction are needed^68^. The combined knowledge of the physicochemical properties of the extractable proteins, the structure and chemical properties of the analyzed tissue could provide new ways for optimizing the pulsed electric field parameters such as electric field strength, pulse duration, pulse number, and frequency. Optimized parameters are critical for the further development of this method for molecular harvesting from various tissue types and for detailed molecular profiling.

Molecular harvesting with electroporation, as introduced in this work, is a new concept for tissue molecular profiling. The feasibility of permeabilization by electroporation is established either to deliver molecules to tissues and cells (drugs, vaccines, etc.) or to directly kill cells^25^. Temporary permeabilization of tissue to facilitate molecular harvesting has not been proposed until this work and to the best of our knowledge, devices that allow for the harvesting of molecules from tissues do not exist. In this study, we show that e-harvesting allows for the extraction of tissue-specific markers from excised tissue samples. This approach could potentially be used for molecular biopsy when the exact location of the tumor is not known. Future studies are needed to develop devices and methods for multiple probing of tissues and tumors in a single multipart procedure. Multiple probing will enable precise molecular cartography of physiological regions, which, in turn, could provide new essential information related to tumor heterogeneity, paving new roads for personalized medicine.

## Methods

### Animals

All animal procedure was approved by the Israel National Council for Animal Experimentation (Israel Ministry of Health number IL-18-3-92). Eight, 8-week old female Athymic Nude mice weighting ~20 g were provided by the Science in Action Ltd. CRO. Five animals were used for the HepG2 model and 3 additional animals were used for the differential expression comparison between normal tissues. The animals were housed in cages with access to food and water ad libitum and were maintained on a 12 h light/dark cycle in a room temperature of around 21 °C and a relative humidity range of 30 to 70%. All *in vivo* experiments were conducted by a professional veterinary in accordance with the Israel National Council for Animal Experimentation guidelines and regulations.

### *In vivo* human HepG2 liver tumor model

Human HepG2 cell line was purchased from ATCC. The cells were grown on the base medium for this cell line (ATCC-formulated Eagle’s Minimum Essential Medium, Catalog No. 30-2003) supplemented with a fetal bovine serum to a final concentration of 10%. After 5 passages, approximately 2 weeks of cultivation, 10^6^ HepG2 cells (50 µL) were directly injected into the mice liver lobe during surgery, similar to^69^. In brief, the animals were anesthetized with Ketamine/Xylazine. The abdomen was shaved and the skin was cleaned with ethanol (70%). The small incision was made on the skin up to the liver. One lobe was exposed and the cells were injected with 0.5 mL syringe and 29G needle. After the injection the skin was sutured with 0/5 thread. Four to five weeks after the injection of the cells, the mice were euthanized with CO_2_ and the tissues were immediately harvested for extraction with pulsed electric fields. The tumor was induced in 5 animals.

### Histology

Specimens were harvested immediately after the treatment and fixed in 10% formalin. Samples in plastic cassettes were dehydrated through ascending ethanol concentrations, transferred into xylene and then paraffinized into paraffin, by an automated machine. Next, the samples were manually embedded into paraffin blocks. The paraffin blocks were sectioned at approximately 3–5 microns thickness. Sections were put on glass slides. Slides were stained with Hematoxylin & Eosin (H&E) and covered by an automated machine.

### Immunohistochemistry

Paraffin blocks were sectioned at approximately 3–5 microns thickness. Sections were put on Super Frost plus glass slides. Slides were incubated overnight at 60 °C. Slides were stained using the standard procedure in Ventana BenchMark Ultra automated slides stainer in combination with Ventana ultra view Universal DAB Detection Kit (Ventana, Roche Diagnostics cat #760-500).

The slides were stained with the following antibodies: monoclonal mouse anti-Human Ki-67, clone MIB-1 (Dako, cat# M7240), diluted 1:200 and monoclonal mouse anti-Human Glypican-3 (GPC3), clone 1G12 (BioCare Medical, cat# PM396 AA), Ready-to-use. Slides were counterstained in Mayer’s Hematoxylene, dehydrated through ascending ethanol concentrations, cleared in Xylene, mounted and covered.

### Pulsed electric field application for biomolecules extraction *ex vivo*

First, 250–300 mg of tissue was excised and loaded into electroporation cuvette (BTX electroporation cuvettes plus, 2 mm, Model No. 620, Harvard Apparatus, MA). The cuvette was inserted into a custom- made electroporation cuvette holder and connected to the electric field pulse generator, (BTX830, Harvard Apparatus, MA). Electroporation was performed using a combination of high-voltage short pulses with low-voltage long pulses, which was shown to increase the efficiency and area of permeabilized tissues^70–72^, as follows: 50 pulses 500 V cm^−1^, 30 µs, 1 Hz, and 50 pulses 50V cm^−1^, 10 ms, delivered at 1 Hz. After the PEF treatment, 300 µl Nuclease-free water was added to the cuvette for “juice” dilution and then liquids transferred to 1.5 ml tubes.

### RNA isolation and amplification from the pulsed electric field extracted juice

#### Normal mouse liver and normal mouse kidney

All samples were collected in fresh conditions and transferred on ice from the surgery room to the laboratory. The extract from 3 mice tissues was separated to 27 normal liver and 18 normal kidney samples after the RNA extraction.

The total RNA was extracted using Water-saturated phenol and 1-Bromo-3-chloropropane (Biological Industries, Beit Haemek Ltd). The cDNA used for PCR was synthesized from total RNA using the GoScript™ Reverse Transcription System (Promega Corporation, Madison, WI, USA).

To select genes for PCR profiling we downloaded mouse liver and kidney RNA-seq data from Newman *et al*. (2017. PMID:28877458) (GEO ID: GSE101657)^60^, which covers five mice per tissue type. Normalization and differential expression (DE) analysis were done using DESeq2^73^. We considered a gene to be DE if it has a corrected p-value < 0.01 and log2(fold-change) > |1| and if it also has average read coverage >100 normalized reads. The selected DE genes: Slc34a1, Umod, Tmem27, Apoa5, F12, and Abcb11 were also manually checked to see if their human orthologs are also liver/kidney-specific according to the human protein atlas (https://www.proteinatlas.org/). To measure expression differences between normal tissues (kidney vs liver) we used PCR with 6 pairs of specific primers for the selected genes. The primers were designed according to the mouse transcriptome (Table S13).

The PCR amplification protocol was 95 °C for 30 s, 40 cycles of 95 °C for 5 s, 55 °C for 10 s, and 72 °C for 30 s.

#### HepG2 cells compared to normal mouse liver

To select genes for comparative PCR measurements in HepG2 vs normal murine liver we downloaded RNA-seq of HepG2 cellular carcinoma and matched normal samples from TCGA (TCGA LIHC, PMID: 28622513)^74^. Normalization and DE analysis were done using DESeq2. We considered a gene as DE, if it has corrected p-value < 0.01 and log2(fold-change) > |1|. The list of genes considered as up-regulated in cancer (the genes with log2(fold-change) > 1) was further filtered to include only genes for which in both HepG2 RNA-seq data from Solomon *et al*. (2017, PMID:29129909)^61^ and HepG2 RNA-seq data from the ENCODE project^62^, an expression level higher than 75% of the expressed genes is observed (reads per kilobase million > 10 (RPKM > 10) in both studies). Using the human protein atlas, we manually checked that the selected genes are considered as elevated in cancer but underexpressed in normal liver. The selected genes were PLK1, TMED3, TMSB10, S100P, and KIF23. To measure expression differences between tumor and normal liver tissue we used PCR with 5 pairs of specific primers for the selected genes. The primers were designed according to the human transcriptome (Table S14).

The PCR amplification protocol was 95 °C for 30 s, 40 cycles of 95 °C for 5 s, 55 °C for 10 s, and 72 °C for 30 s Table S14. Primers used for the mouse liver and human HepG2 tumor in mouse liver differentiation by the RNA extracted with electroporation.

All samples were collected in fresh conditions and transferred on ice from the surgery room to the laboratory. The extract from 5 mice tissues was separated into 7 tumors and 14 normal kidney samples after the RNA extraction. The RNA was separated using 1.2% E-Gel electrophoreses system (ThemoFisher, CA). The gel images were captured with ENDURO^TM^ GDS camera (Labnet Inc., NJ). Quantification was done with ImageJ (ver 1.52e, NIH, MA).

### Isolating proteins from the pulsed electric field extracted juice

Proteins were isolated from the PEF extract using the protocol of EZ- RNA II kit (Biological Industries, Beit Haemek Ltd). Air-dried protein pellets were taken for proteomic analysis as described below.

### Identifying and quantifying proteins using LC-MS/MS

#### Proteolysis

The samples were brought to 8 M urea, 400 mM ammonium bicarbonate, 10 mM DTT, vortexed, sonicated for 5′ at 90% with 10–10 cycles, and centrifuged. Protein amount was estimated using Bradford readings. 20 ug protein from each sample was reduced 60 °C for 30 min, modified with 37.5 mM iodoacetamide in 400 mM ammonium bicarbonate (in the dark, room temperature for 30 min) and digested in 2 M Urea, 100 mM ammonium bicarbonate with modified trypsin (Promega) at a 1:50 enzyme-to-substrate ratio, overnight at 37 °C. Additional second digestion with trypsin was done for 4 hours at 37 °C.

#### Mass spectrometry analysis

The tryptic peptides were desalted using C18 tips (Harvard) dried and re-suspended in 0.1% Formic acid. The peptides were resolved by reverse-phase chromatography on 0.075 × 180-mm fused silica capillaries (J&W) packed with Reprosil reversed-phase material (Dr. Maisch GmbH, Germany). The peptides were eluted with linear 180 minutes gradient of 5 to 28% 15 minutes gradient of 28 to 95% and 25 minutes at 95% acetonitrile with 0.1% formic acid in water at flow rates of 0.15 μl/min. Mass spectrometry was performed by Q-Exactive Plus mass spectrometer (Thermo) in a positive mode using repetitively full MS scan followed by collision induces dissociation (HCD) of the 10 most dominant ions selected from the first MS scan.

The mass spectrometry data from all the biological repeats were analyzed using the MaxQuant software 1.5.2.8 (Mathias Mann’s group) vs. the mouse proteome from the UniProt database with 1% FDR. The data were quantified by label-free analysis using the same software, based on extracted ion currents (XICs) of peptides enabling quantitation from each LC/MS run for each peptide identified in any of the experiments.

### Statistical analysis

We statistically analyzed the functional groups of the extracted proteins using Gene Ontology (GO) analysis with GOrilla, annotating the ranked gene list to the mouse genome. GOrilla is based on the mHG statistics^42,48,75^.

Comparison of ranked lists: m^2^HG. To compare two ranked lists we compute the relative permutation of one against the other and then assess the significance of the size of the intersection of two prefixes of the lists by using a hypergeometric model. The results are corrected for multiple testing. For details and implementations see literature that describes the methodology^46–48^.

Distribution fits and comparative statistical analysis of molecular properties was performed using R-studio for Windows. Differential expression p-values were calculated using one-sided Student t-test using R as well as Matlab and Python implementations.

## Supplementary information

Supplementary information

Table S1 LiverProteomics

Table S2 KidneyProteomics

Table S3 LiveVsKidneyProteomics

Table S4 LiverVsKidney Process

Table S5 LiverVsKidneyFunction

Table S6LiverVsKidneyComponent

Table S7LiverKidneyVSLiterautreProteomics

Table S8HepG2Proteomics

Table S9 TumorLiverLFQ

Table S10 TumorVsLiverProcess

Table S11 TumorVsLiverFunction

Table S12 TumorVsLiverComponent

## Data Availability

The authors declare that all data supporting the findings of this study are available within the paper and its Supplementary Information.

## References

[1] Chapman PB (2011). Improved survival with vemurafenib in melanoma with BRAF V600E mutation. N Eng J Med.

[2] Chan BA, Hughes BG (2015). Targeted therapy for non-small cell lung cancer: current standards and the promise of the future. Transl Lung Cancer Res.

[3] Ellis PM, Coakley N, Feld R, Kuruvilla S, Ung YC (2015). Use of the epidermal growth factor receptor inhibitors gefitinib, erlotinib, afatinib, dacomitinib, and icotinib in the treatment of non-small-cell lung cancer: a systematic review. Curr. Oncol..

[4] Bittner M (2000). Molecular classification of cutaneous malignant melanoma by gene expression profiling. Nature.

[5] Hedenfalk I (2001). Gene-Expression Profiles in Hereditary Breast Cancer. N. Engl. J. Med..

[6] Haakensen VD (2016). Serum N-glycan analysis in breast cancer patients - Relation to tumour biology and clinical outcome. Mol. Oncol..

[7] Golub, T. R. *et al*. Molecular classification of cancer: Class discovery and class prediction by gene expression monitoring. *Science* (*80-*)., 10.1126/science.286.5439.531 (1999). 10.1126/science.286.5439.531 10521349

[8] Ludwig JA, Weinstein JN (2005). Biomarkers in cancer staging, prognosis and treatment selection. Nature Reviews Cancer.

[9] Massuti B, Sanchez JM, Hernando-Trancho F, Karachaliou N, Rosell R (2013). Are we ready to use biomarkers for staging, prognosis and treatment selection in early-stage non-small-cell lung cancer. Transl. lung cancer Res..

[10] Heitzer, E., Haque, I. S., Roberts, C. E. S. & Speicher, M. R. Current and future perspectives of liquid biopsies in genomics-driven oncology. *Nature Reviews Genetics*, 10.1038/s41576-018-0071-5 (2019). 10.1038/s41576-018-0071-5 30410101

[11] Alieva, M. *et al*. Preventing inflammation inhibits biopsy-mediated changes in tumor cell behavior. *Sci*. *Rep*., 10.1038/s41598-017-07660-4 (2017). 10.1038/s41598-017-07660-4 PMC5548904 28790339

[12] Exner GU, Kurrer MO, Mamisch-Saupe N, Cannon SR (2017). The tactics and technique of musculoskeletal biopsy. Efort Open Rev..

[13] Mathenge, E. G. *et al*. Core Needle Biopsy of Breast Cancer Tumors Increases Distant Metastases in a Mouse Model. *Neoplasia*, 10.1016/j.neo.2014.09.004 (2014). 10.1016/j.neo.2014.09.004 PMC4240917 25425969

[14] Sennerstam, R. B., Franzén, B. S. H., Wiksell, H. O. T. & Auer, G. U. Core-needle biopsy of breast cancer is associated with a higher rate of distant metastases 5 to 15 years after diagnosis than FNA biopsy. *Cancer Cytopathol*., 10.1002/cncy.21909 (2017). 10.1002/cncy.21909 28837268

[15] Alieva M, van Rheenen J, Broekman MLD (2018). Potential impact of invasive surgical procedures on primary tumor growth and metastasis. Clin. Exp. Metastasis.

[16] Tosoian, J. J. & Antonarakis, E. S. Molecular heterogeneity of localized prostate cancer: more different than alike. *Transl*. *Cancer Res*. *Vol* 6, *Suppl*. 1 (*February* 2017) *Transl*. *Cancer Res*., 10.21037/tcr.2017.02.17 (2017). 10.21037/tcr.2017.02.17 PMC5438201 28529909

[17] Wei L (2017). Intratumoral and Intertumoral Genomic Heterogeneity of Multifocal Localized Prostate Cancer Impacts Molecular Classifications and Genomic Prognosticators. Eur. Urol..

[18] Gerlinger M (2012). Intratumor heterogeneity and branched evolution revealed by multiregion sequencing. N. Engl. J. Med..

[19] Tannock IF, Hickman JA (2016). Limits to Personalized Cancer Medicine. N. Engl. J. Med..

[20] Ellsworth RE, Blackburn HL, Shriver CD, Soon-Shiong P, Ellsworth DL (2017). Molecular heterogeneity in breast cancer: State of the science and implications for patient care. Semin. Cell Dev. Biol..

[21] Thomsen, M. B. H. *et al*. Comprehensive multiregional analysis of molecular heterogeneity in bladder cancer. *Sci*. *Rep*. **7** (2017). 10.1038/s41598-017-11291-0 PMC5600970 28916750

[22] Dagogo-Jack I, Shaw AT (2018). Tumour heterogeneity and resistance to cancer therapies. Nature Reviews Clinical Oncology.

[23] Ofiara, L. M., Navasakulpong, A., Beaudoin, S. & Gonzalez, A. V. Optimizing Tissue Sampling for the Diagnosis, Subtyping, and Molecular Analysis of Lung Cancer. *Front*. *Oncol*. **4** (2014). 10.3389/fonc.2014.00253 PMC4170137 25295226

[24] Cyll K (2017). Tumour heterogeneity poses a significant challenge to cancer biomarker research. Br. J. Cancer.

[25] Yarmush ML, Golberg A, Serša G, Kotnik T, Miklavčič D (2014). Electroporation-Based Technologies for Medicine: Principles, Applications, and Challenges. Annu. Rev. Biomed. Eng..

[26] Bale SS (2015). Long-Term Coculture Strategies for Primary Hepatocytes and Liver Sinusoidal Endothelial Cells. Tissue Eng. Part C Methods.

[27] Rubinsky, B. Electrical Field and Temperature Model of Nonthermal Irreversible Electroporation in Heterogeneous Tissues. *J*. *Biomech*. *Eng*., 10.1115/1.3156808 (2009). 10.1115/1.3156808 19640131

[28] Golberg, A., Fischer, J. & Rubinsky, B. The Use of Irreversible Electroporation in Food Preservation. *Irreversible Electroporation* 273–312 (2010).

[29] Goldberg A, Rubinsky B (2010). The effect of electroporation type pulsed electric fields on DNA in aqueous solution. Technol. Cancer Res. Treat..

[30] Golberg A, Rubinsky B (2012). Towards electroporation based treatment planning considering electric field induced muscle contractions. Technol. Cancer Res. Treat..

[31] Kos B, Voigt P, Miklavcic D, Moche M (2015). Careful treatment planning enables safe ablation of liver tumors adjacent to major blood vessels by percutaneous irreversible electroporation (IRE). Radiol. Oncol..

[32] Garcia PA, Davalos RV, Miklavcic D (2014). A Numerical Investigation of the Electric and Thermal Cell Kill Distributions in Electroporation-Based Therapies in Tissue. PLoS One.

[33] Groselj A (2015). Coupling treatment planning with navigation system: a new technological approach in treatment of head and neck tumors by electrochemotherapy. Biomed. Eng. Online.

[34] Zupanic, A., Kos, B. & Miklavcic, D. Treatment planning of electroporation-based medical interventions: Electrochemotherapy, gene electrotransfer and irreversible electroporation. *Phys*. *Med*. *Biol*., 10.1088/0031-9155/57/17/5425 (2012). 10.1088/0031-9155/57/17/5425 22864181

[35] Pavliha, D., Mušič, M. M., Serša, G. & Miklavčič, D. Electroporation-Based Treatment Planning for Deep-Seated Tumors Based on Automatic Liver Segmentation of MRI Images. *PLoS One*, 10.1371/journal.pone.0069068 (2013). 10.1371/journal.pone.0069068 PMC3732275 23936315

[36] Polikovsky M (2016). Towards marine biorefineries: Selective proteins extractions from marine macroalgae Ulva with pulsed electric fields. Innov. Food Sci. Emerg. Technol..

[37] Robin A (2018). Deashing macroalgae biomass by pulsed electric field treatment. Bioresour. Technol..

[38] Polikovsky M (2019). *In silico* food allergenic risk evaluation of proteins extracted from macroalgae Ulva sp. with pulsed electric fields. Food Chem..

[39] Edd, J. F., Horowitz, L., Davalos, R. V., Mir, L. M. & Rubinsky, B. *In vivo* results of a new focal tissue ablation technique: Irreversible electroporation. *IEEE Trans*. *Biomed*. *Eng*., 10.1109/TBME.2006.873745 (2006). 10.1109/TBME.2006.873745 16830945

[40] Golberg, A., Bruinsma, B. G., Jaramillo, M., Yarmush, M. & Uygun, B. E. Rat liver regeneration following ablation with irreversible electroporation. *PeerJ* (2016). 10.7717/peerj.1571 PMC4727979 26819842

[41] Djokic, M. *et al*. Electrochemotherapy as treatment option for hepatocellular carcinoma, a prospective pilot study. *Eur*. *J*. *Surg*. *Oncol*., 10.1016/j.ejso.2018.01.090 (2018). 10.1016/j.ejso.2018.01.090 29402556

[42] Eden, E., Navon, R., Steinfeld, I., Lipson, D. & Yakhini, Z. GOrilla: A tool for discovery and visualization of enriched GO terms in ranked gene lists. *BMC Bioinformatics* **10** (2009). 10.1186/1471-2105-10-48 PMC2644678 19192299

[43] Puisac, B. *et al*. Differential HMG-CoA lyase expression in human tissues provides clues about 3-hydroxy-3-methylglutaric aciduria. *J*. *Inherit*. *Metab*. *Dis*., 10.1007/s10545-010-9097-3 (2010). 10.1007/s10545-010-9097-3 PMC2903694 20532825

[44] Saksela, M., Lapatto, R. & Raivio, K. O. Xanthine oxidoreductase gene expression and enzyme activity in developing human tissues. *Biol*. *Neonate*, 10.1159/000014034 (1998). 10.1159/000014034 9701649

[45] Huttlin, E. L. *et al*. A tissue-specific atlas of mouse protein phosphorylation and expression. *Cell*, 10.1016/j.cell.2010.12.001 (2010). 10.1016/j.cell.2010.12.001 PMC3035969 21183079

[46] Cohn-Alperovich, D., Rabner, A., Kifer, I., Mandel-Gutfreund, Y. & Yakhini, Z. Mutual enrichment in aggregated ranked lists with applications to gene expression regulation. In *Bioinformatics*, 10.1093/bioinformatics/btw435 (2016). 10.1093/bioinformatics/btw435 27587663

[47] Leibovich, L. & Yakhini, Z. Efficient motif search in ranked lists and applications to variable gap motifs. *Nucleic Acids Res*., 10.1093/nar/gks206 (2012). 10.1093/nar/gks206 PMC3401424 22416066

[48] Enerly, E. *et al*. miRNA-mRNA integrated analysis reveals roles for mirnas in primary breast tumors. *PLoS One* **6** (2011). 10.1371/journal.pone.0016915 PMC3043070 21364938

[49] Vongchan, P. & Linhardt, R. J. Characterization of a new monoclonal anti-glypican-3 antibody specific to the hepatocellular carcinoma cell line, HepG2. *World J*. *Hepatol*., 10.4254/wjh.v9.i7.368 (2017). 10.4254/wjh.v9.i7.368 PMC5340992 28321273

[50] Yang, C. *et al*. Marker of proliferation Ki-67 expression is associated with transforming growth factor beta 1 and can predict the prognosis of patients with hepatic B virus-related hepatocellular carcinoma. *Cancer Manag*. *Res*., 10.2147/CMAR.S162595 (2018). 10.2147/CMAR.S162595 PMC5901156 29692627

[51] Tan, S. C. & Yiap, B. C. DNA, RNA, and protein extraction: The past and the present. *Journal of Biomedicine and Biotechnology*, 10.1155/2009/574398 (2009). 10.1155/2009/574398 PMC2789530 20011662

[52] Cañas, B., Piñeiro, C., Calvo, E., López-Ferrer, D. & Gallardo, J. M. Trends in sample preparation for classical and second generation proteomics. *Journal of Chromatography A*, 10.1016/j.chroma.2007.01.045 (2007). 10.1016/j.chroma.2007.01.045 17276441

[53] W. Burden, D. Guide to the Disruption of Biological Samples. *Random Prim*., 10.1111/j.1474-9726.2006.00237.x (2012).

[54] Raynie, D. E. Modern extraction techniques. *Anal*. *Chem*., 10.1021/ac101223c (2010). 10.1021/ac101223c 20481433

[55] Ali, N., Rampazzo, R. D. C. P., Costa, A. Di. T. & Krieger, M. A. Current Nucleic Acid Extraction Methods and Their Implications to Point-of-Care Diagnostics. *BioMed Research International*, 10.1155/2017/9306564 (2017). 10.1155/2017/9306564 PMC5529626 28785592

[56] Feist, P. & Hummon, A. B. Proteomic challenges: Sample preparation techniques for Microgram-Quantity protein analysis from biological samples. *International Journal of Molecular Sciences*, 10.3390/ijms16023537 (2015). 10.3390/ijms16023537 PMC4346912 25664860

[57] Geng, T., Bao, N., Sriranganathanw, N., Li, L. & Lu, C. Genomic DNA extraction from cells by electroporation on an integrated microfluidic platform. *Anal*. *Chem*., 10.1021/ac3026064 (2012). 10.1021/ac3026064 PMC3492889 23061629

[58] Bahi, M. M., Tsaloglou, M. N., Mowlem, M. & Morgan, H. Electroporation and lysis of marine microalga Karenia brevis for RNA extraction and amplification. *J*. *R*. *Soc*. *Interface*, 10.1098/rsif.2010.0445 (2011). 10.1098/rsif.2010.0445 PMC3061125 21084344

[59] Ghosh, S., Gillis, A., Sheviryov, J., Levkov, K. & Golberg, A. Towards waste meat biorefinery: Extraction of proteins from waste chicken meat with non-thermal pulsed electric fields and mechanical pressing. *J*. *Clean*. *Prod*., 10.1016/J.JCLEPRO.2018.10.037 (2018).

[60] Newman, J. C. *et al*. Ketogenic Diet Reduces Midlife Mortality and Improves Memory in Aging Mice. *Cell Metab*., 10.1016/j.cmet.2017.08.004 (2017). 10.1016/j.cmet.2017.08.004 PMC5605815 28877458

[61] Solomon, O. *et al*. RNA editing by ADAR1 leads to context-dependent transcriptome-wide changes in RNA secondary structure. *Nat*. *Commun*. **8** (2017). 10.1038/s41467-017-01458-8 PMC5682290 29129909

[62] Dunham, I. *et al*. An integrated encyclopedia of DNA elements in the human genome. *Nature*, 10.1038/nature11247 (2012). 10.1038/nature11247 PMC3439153 22955616

[63] Fleige, S. & Pfaffl, M. W. RNA integrity and the effect on the real-time qRT-PCR performance. *Molecular Aspects of Medicine*, 10.1016/j.mam.2005.12.003 (2006). 10.1016/j.mam.2005.12.003 16469371

[64] Golberg, A. The impact of pulsed electric fields on cells and biomolecules. Comment on ‘Lightning-triggered electroporation and electrofusion as possible contributors to natural horizontal gene transfer’ by Tadej Kotnik, 10.1016/j.plrev.2013.07.025 (2013). 10.1016/j.plrev.2013.07.025 23948139

[65] Peirson, S. N. & Butler, J. N. RNA extraction from mammalian tissues. *Methods Mol*. *Biol*., 10.1385/1-59745-257-2:315 (2007). 10.1007/978-1-59745-257-1_22 17417019

[66] Golberg A (2016). Energy Efficient Biomass Processing with Pulsed Electric Fields for Bioeconomy and Sustainable Development. Biotechnol. Biofuels.

[67] Ngoka, L. C. M. Sample prep for proteomics of breast cancer: Proteomics and gene ontology reveal dramatic differences in protein solubilization preferences of radioimmunoprecipitation assay and urea lysis buffers. *Proteome Sci*., 10.1186/1477-5956-6-30 (2008). 10.1186/1477-5956-6-30 PMC2600628 18950484

[68] Ericsson, C. & Nister, M. Protein extraction from solid tissue. *Methods Mol Biol*, https://doi.org/10.1007/978-1-59745-423-0{\textunderscore}17 (2011). 10.1007/978-1-59745-423-0_17 20949398

[69] Woodfield, S. E. *et al*. A Novel Cell Line Based Orthotopic Xenograft Mouse Model That Recapitulates Human Hepatoblastoma. *Sci*. *Rep*., 10.1038/s41598-017-17665-8 (2017). 10.1038/s41598-017-17665-8 PMC5736579 29259231

[70] Yao C (2017). Synergistic combinations of short high-voltage pulses and long low-voltage pulses enhance irreversible electroporation efficacy. Sci. Rep..

[71] Yao, C., Lv, Y., Dong, S., Zhao, Y. & Liu, H. Irreversible electroporation ablation area enhanced by synergistic high-and low-voltage pulses. *PLoS One*, 10.1371/journal.pone.0173181 (2017). 10.1371/journal.pone.0173181 PMC5333894 28253331

[72] Andre F (2008). Efficiency of high and low voltage pulse combinations for gene electrotransfer in muscle, liver, tumor and skin. Hum. Gene Ther..

[73] Love, M. I., Huber, W. & Anders, S. Moderated estimation of fold change and dispersion for RNA-seq data with DESeq2. *Genome Biol*., 10.1186/s13059-014-0550-8 (2014). 10.1186/s13059-014-0550-8 PMC4302049 25516281

[74] Ally, A. *et al*. Comprehensive and Integrative Genomic Characterization of Hepatocellular Carcinoma. *Cell*, 10.1016/j.cell.2017.05.046 (2017). 10.1016/j.cell.2017.05.046 PMC5680778 28622513

[75] Steinfeld, I., Navon, R., Ardigò, D., Zavaroni, I. & Yakhini, Z. Clinically driven semi-supervised class discovery in gene expression data. In *Bioinformatics*, 10.1093/bioinformatics/btn279 (2008). 10.1093/bioinformatics/btn279 18689846

