# Supplementary material for: Molecular harvesting with electroporation for tissue profiling
Source: Sci Rep. 2019 Oct 31;9:15750. doi: 10.1038/s41598-019-51634-7 (PMC6823461; doi:10.1038/s41598-019-51634-7)
Supplement: Supplementary file 1 — Supplementary information [file 41598_2019_51634_MOESM1_ESM.pdf]

Molecular harvesting with electroporation for tissue profiling.

Alexander Golberg[1]*, Julia Sheviryov[1], Oz Solomon[2], Leon Anavy[3], Zohar Yakhini[2,3]**.

[1] Porter School of Environment and Earth Sciences, Tel Aviv University, Tel Aviv, Israel.

[2] School of Computer Science, Herzliya Interdisciplinary Center, Herzliya, Israel.

[3] Computer Science Department, Technion, Haifa, Israel.

Supplementary information

Analysis of the molecular weight of the extracted with electroporation proteins from mouse liver and kidney.

Using semi-quantitative proteomic data, we calculated the molecular weight (MW), the normalized intensity for each sample (LFQ), intensity and normalized within sample intensity (iBAQ, **Fig. S 2,3a**) for proteins extracted from the liver (**Table S1**) and the kidney (**Table S2**). Using these quantitative data, we selected the list of most abundant proteins with iBAQ>10^7 for further analysis. The histogram and density function (**Fig. S3 b,c,d, S4 b,c,d**) suggested the e-harvested proteins have a not-normal and skewed to the write distribution function. The skewness and kurtosis plots of MW (**Fig. S3e, S4e**) suggested that MW has lognormal, gamma or Weibull distributions. Histogram of the fitted densities, Q-Q plot, CDF and P-P plots of these three distributions appear in **Fig. S3 f, g, h, i, and Fig 4f, g, h, i** respectively. The goodness of fit analysis (**Table S15, S16**) suggests that MW of the most abundant proteins extracted by electroporation is closer to lognormal distribution (smallest statistics for all checked criteria) (**Table S17, S18**). The parameters and the uncertainty in the parameters (confidence interval) for the lognormal distribution function were determined using bootstrapping [1].

Interesting, the proteins extracted from the kidney had almost twice smaller MW than the proteins extracted from the liver. This can be explained by a different electroporation threshold of cells and by different diffusion properties of properties in these two media[2].

Analysis of the molecular weight of the extracted with electroporation proteins from the HepG2 tumor.

Using semi-quantitative proteomic data, we calculated the molecular weight (MW), the normalized intensity for each sample (LFQ), and intensity and normalized within sample intensity (iBAQ, **Fig. S5a**) for proteins extracted from the HepG2 tumor (**Table S8**). Using these quantitative data, we selected the list of most abundant proteins with iBAQ>10^7 for further analysis. The histogram and density functions (**Fig. S5 b, c, d**) suggested the e-harvested proteins have a not-normal and skewed to the write distribution function. The skewness and kurtosis plots of MW (**Fig. S5e**) suggested that MW has lognormal, gamma or Weibull distributions. Histogram of the fitted densities, Q-Q plot, CDF and P-P plots of these three distributions appear in **Fig. S5 f, g, h, i** respectively. The goodness of fit analysis (**Table S19**) suggests that MW of the most abundant proteins extracted by electroporation is closer to lognormal distribution (smallest statistics for all checked criteria) (**Table S20**).

Supplementary references

1. Efron, B.; Tibshirani, R. *An Introduction to the Bootstrap*; 1993.

2. Golberg, A.; Rubinsky, B. Mass transfer phenomena in Electroporation. In *Transport in Biological Media*; Elsevier, 2013; pp. 456–492.

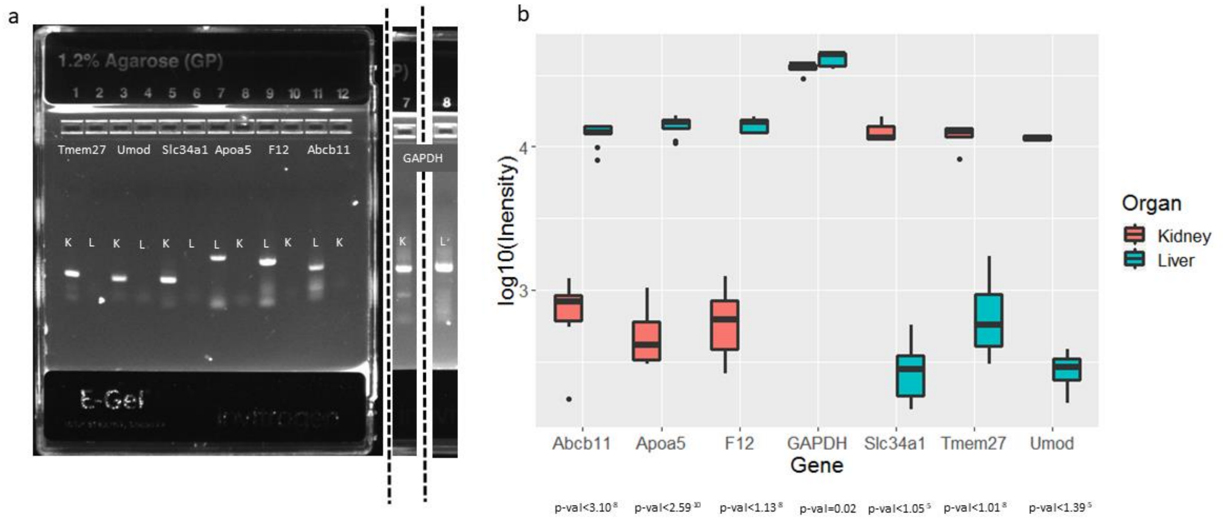

Figure S1. Differential expression of genes detected with RNA extracted with electroporation molecular harvesting in mouse liver and kidney N=6. **a**. Gel digital image. GAPDH was run on separate gels. **b**. Quantification of a signal.

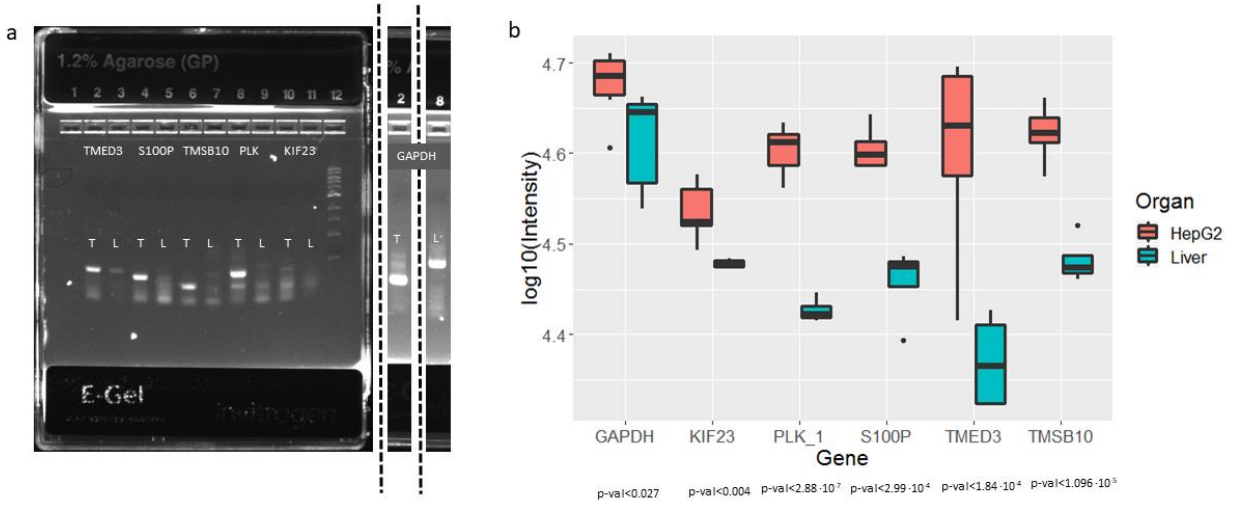

Figure S2. Differential expression of genes detected with RNA extracted using the proposed e-harvesting protocol from mouse liver and from HepG2. N=6. **a**. Gel digital image. GAPDH was run on separate gels. **b**. Quantification of a signal.

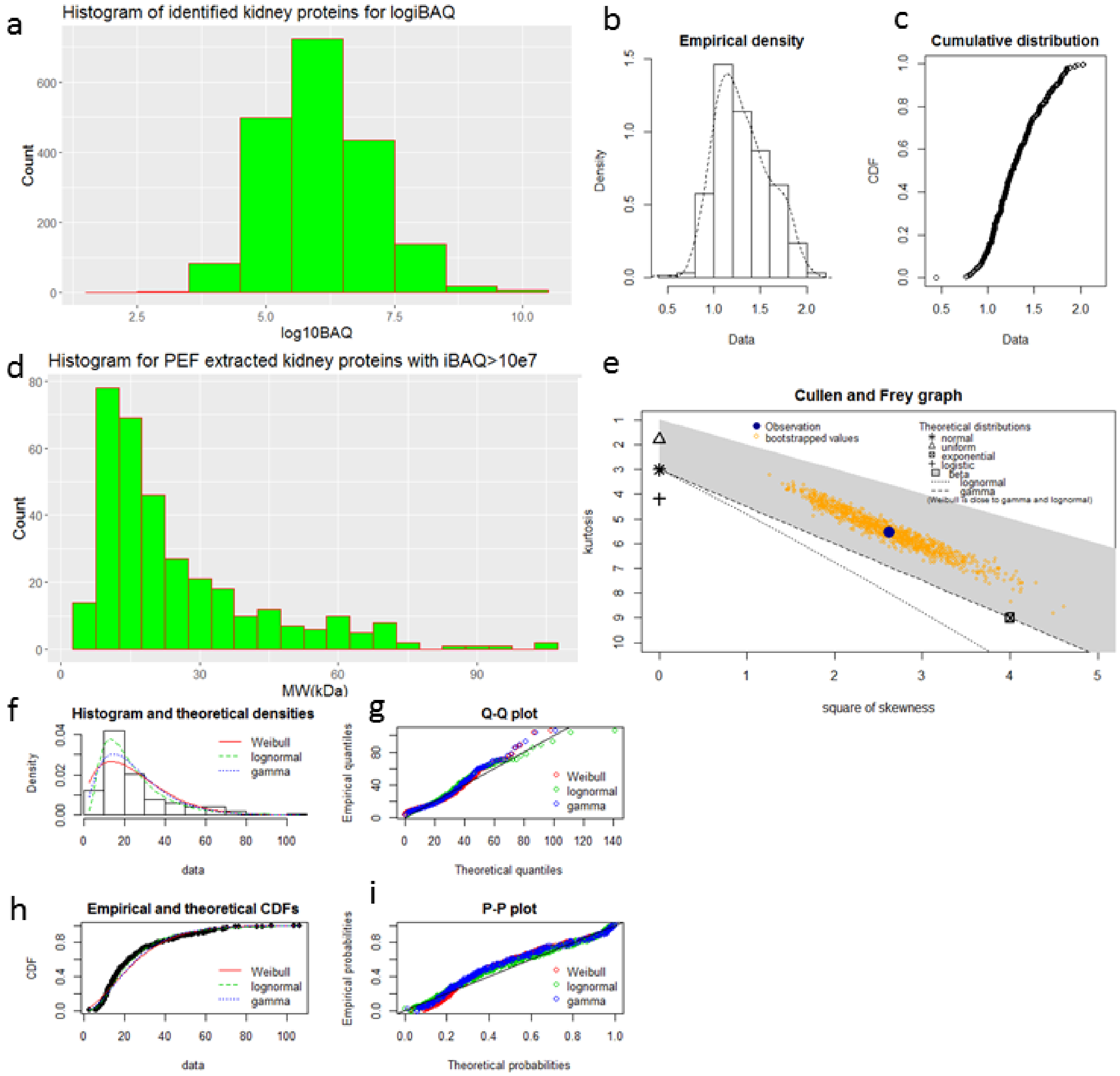

Figure S3. Molecular weights (MW) of proteins extraction with electroporation from a normal mouse liver. **a**. Histogram of MW of all extracted and identified proteins. **b**. Density function and **c**. Cumulative distribution of MW of the extractable proteins. **d**. Histogram of MW of extracted and identified proteins with iBAQ>10^7. **e**. Cullen and Frey graph for MW distribution analysis. f. Histogram of fitted densities. g. Q-Q plot of the fitted molecular weight distributions. h. CFD of the fitted molecular weight distributions. i. Q-Q plot of the fitted molecular weight distributions.

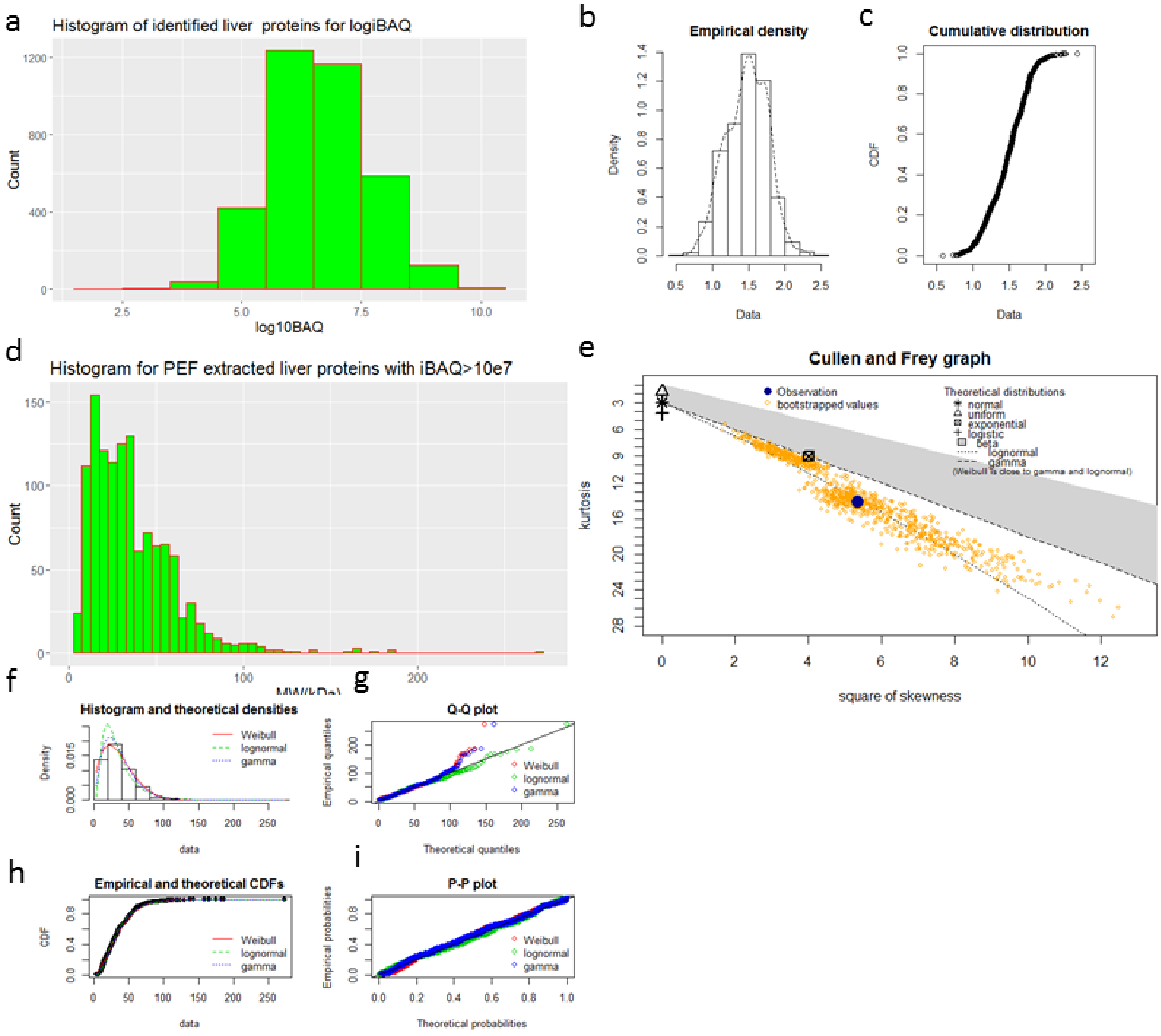

Figure S4. Molecular weights (MW) of proteins extraction with electroporation from a normal mouse kidney. **a**. Histogram of MW of all extracted and identified proteins. **b**. Density function and **c**. Cumulative distribution of MW of the extractable proteins. **d**. Histogram of MW of extracted and identified proteins with iBAQ>10^7. **e**. Cullen and Frey graph for MW distribution analysis. f. Histogram of fitted densities. g. Q-Q plot of the fitted molecular weight distributions. h. CFD of the fitted molecular weight distributions. i. Q-Q plot of the fitted molecular weight distributions.

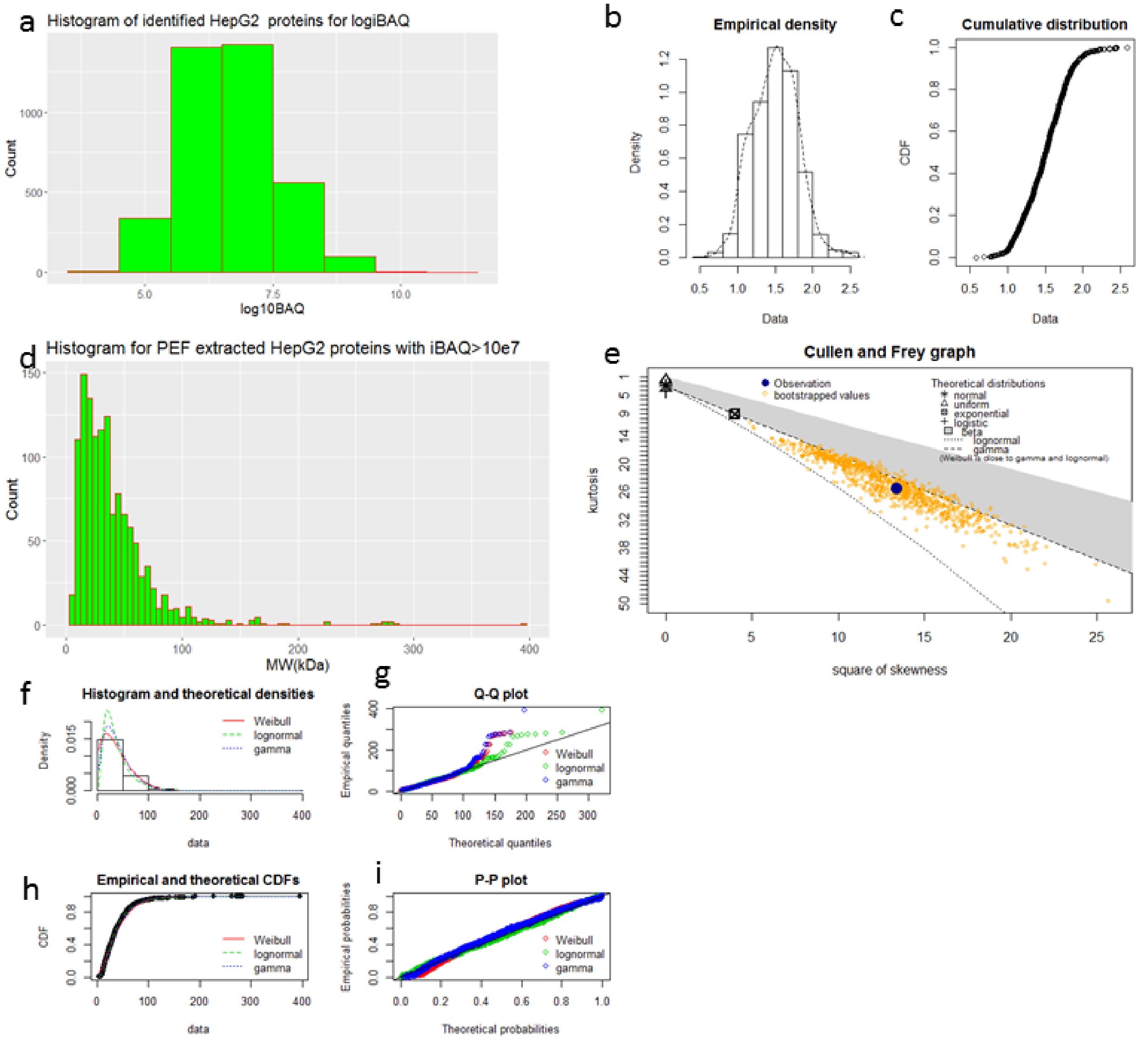

Figure S5. Molecular weights (MW) of proteins extraction with electroporation from a HepG2 tumor model in the mouse liver. **a**. Histogram of MW of all extracted and identified proteins. **b**. Density function and **c**. Cumulative distribution of MW of the extractable proteins. **d**. Histogram of MW of extracted and identified proteins with iBAQ>10^7. **e**. Cullen and Frey graph for MW distribution analysis. f. Histogram of fitted densities. g. Q-Q plot of the fitted molecular weight distributions. h. CFD of the fitted molecular weight distributions. i. Q-Q plot of the fitted molecular weight distributions.

Table S13. Primers used for the mouse liver and mouse kidney differentiation by the RNA extracted with electroporation.

Gene Abbreviation	Gene name	forward/reverse primer
Slc34a1	solute carrier family 34 (sodium phosphate), member 1	5'- GAT GTC CTA CAG CGA GAG ATT G -3'<br>5'- GGG AGC AGA CAA AGA GGT AAA -3'
Umod	uromodulin	5'- TCC CGG TTT GTA CTG CTA ATG -3'<br>5'- TGG ACA CCT TGT CGT GTT ATG -3'
Tmem27	collectrin, amino acid transport regulator	5'- GTT TGC GGC TCT GAA AGA ATG -3'<br>5'- CAC TGT TGA TCC GGT TCC TAT T -3'
Apoa5	Apolipoprotein A-V	5'- GAC GAC CTG TGG GAA GAT ATT G -3'<br>5'- CAG GAG GTA GGG ACT GTA TGA -3'
F12	coagulation factor XII (Hageman factor	5'- GAG GAA CTG ACA GTG GTA CTT G -3'<br>5'- GGG AAG GAT AAA GCC TGG TTA G -3'
Abcb11	ATP Binding Cassette Subfamily B Member 11	5'- CTG TGG GTT GGT GGA CAT TA -3'<br>5'- GAG AGG ACT TCA TCG GCA ATA G -3'
GAPDH_mouse	Glyceraldehyde 3-phosphate dehydrogenase	5'- GGG TGT GAA CCA CGA GAA ATA -3'<br>5'- GGG TCT GGG ATG GAA ATT GT -3'

Table S14. Primers used for the mouse liver and HepG2 kidney differentiation by the RNA extracted with electroporation.

Gene Abbreviation	Gene name	forward/reverse primer
**PLK1**	Serine/threonine-protein kinase	5'- CAG CAA GTG GGT GGA CTA TT -3'<br>5'- ATC AGT GGG CAC AAG ATG AG -3'
**TMED3**	Transmembrane p24 trafficking protein 3	5'- GAT TGA CTC CCA GAC GCA TTA C -3'<br>5'- CAG TCG GAT GCC TTC TGA TTA C -3'
**TMSB10**	Thymosin beta-10	5'- CGA GAC TGC ACG GAT TGT T -3'<br>5'- CAT CTT GCA GGT GGC TCT T -3'
**S100P**	S100 calcium-binding protein P	5'- AGG AAG GTG GGT CTG AAT CT -3'<br>5'- AGG AAG GTG GGT CTG AAT CT -3'
**KIF23**	Kinesin-like protein KIF23	5'- AGT GTG AGG TTG ATG CCT TAT T -3'<br>5'- CTC TGG TCC GGT TAG TTC TTT C -3'
**GAPDH_mouse**	**Glyceraldehyde 3-phosphate dehydrogenase**	5'- GGG TGT GAA CCA CGA GAA ATA -3'<br>5'- GGG TCT GGG ATG GAA ATT GT -3'
**GAPDH_human**	**Glyceraldehyde 3-phosphate dehydrogenase**	5'- GAT TCC ACC CAT GGC AAA TTC -3'<br>5'- GTC ATG AGT CCT TCC ACG ATA C -3'

Table S15. The goodness of fit analysis of highly abundant electroporation extracted kidney proteins (iBAQ>10^7).

**Goodness-of-fit statistics**			
	**Weibull**	**lognormal**	**gamma**
Kolmogorov-Smirnov statistic	0.1049928	0.06978198	0.1079061
Cramer-von Mises statistic	1.3454622	0.43578147	1.1439954
Anderson-Darling statistic	8.1846425	2.68808789	6.5859668
**Goodness-of-fit criteria**			
Akaike's Information Criterion	2776.322	2706.840	2746.318
Bayesian Information Criterion	2783.968	2714.486	2753.965

Table S16. The goodness of fit analysis of highly abundant electroporation extracted liver proteins (iBAQ>10^7).

**Goodness-of-fit statistics**			
	**Weibull**	**lognormal**	**gamma**
Kolmogorov-Smirnov statistic	0.05698024	0.0374255	0.03459732
Cramer-von Mises statistic	0.84034789	0.4859410	0.36004244
Anderson-Darling statistic	7.69096820	2.9875080	2.82114915
**Goodness-of-fit criteria**			
Akaike's Information Criterion	10940.07	10805.36	10845.93
Bayesian Information Criterion	10950.31	10815.59	10856.16

Table S17. Parametric bootstrap medians and 95% percentile CI for lognormal distribution of the MW of electroporation extracted kidney proteins.

	**Median**	**2.5%**	**97.5%**
**meanlog**	3.0043648	2.9364384	3.0735471
**sdlog**	0.6527423	0.6061976	0.7046646

Table S18. Parametric bootstrap medians and 95% percentile CI for lognormal distribution of the MW of electroporation extracted liver proteins.

	**Median**	**2.5%**	**97.5%**
**meanlog**	3.3893310	3.3525484	3.4260361
**sdlog**	0.6514971	0.6241754	0.6792361

Table S19. The goodness of fit analysis of highly abundant electroporation extracted HepG2 proteins (iBAQ>10^7).

	Weibull	lognormal	gamma
**Goodness-of-fit statistics**			
Kolmogorov-Smirnov statistic	0.08502379	0.02567092	0.05181307
Cramer-von Mises statistic	2.15728759	0.16256876	1.04995599
Anderson-Darling statistic	17.59556665	1.31084115	7.67302556
**Goodness-of-fit criteria**			
Akaike's Information Criterion	11712.26	11440.62	11584.03
Bayesian Information Criterion	11722.56	11450.91	11594.33

Table S20. Parametric bootstrap medians and 95% percentile CI for lognormal distribution of the MW of PEF extracted HepG2 proteins.

	Median	2.5%	97.5%
**meanlog**	3.4474497	3.4095316	3.486965
**sdlog**	0.6928734	0.6671016	0.719039
